# The Physician and the Venereal Peril

**Published:** 1907-12

**Authors:** Stewart R. Roberts

**Affiliations:** Professor of Physiology, Atlanta School of Medicine, Atlanta, Ga.


### THE PHYSICIAN AND THE VENEREAL PERIL.

BY STEWART R. ROBERTS, M. S., M. D., PROFESSOR OF PHYSIOLOGY, ATLANTA SCHOOL OF MEDICINE, ATLANTA, GA.

The far reaching ravages of the Social Evil have been largely overlooked, not only by the medical profession, but by all thinking men. The oversight may be due to the fact that the medical profession has never informed the public as to the true status of affairs. The physicians of today are awake to the dangers of the Social Evil, and the salvation for the future lies in the unselfish work of the medical profession.

After an experience of about three years in speaking and conversing with thousands of college students throughout the Southern States on this subject, I have come to two conclusions: First: the average boy feels that he needs and is anxious to receive instruction on matters pertaining to sex, and the venereal diseases; Secondly: he must be appealed to from a moral point of view, as well as given facts and figures relating to these diseases and their results. We must remember that the Social Evil is a moral problem as well as a social problem, and to leave out the moral issues at stake is to present but half the question To set the morality of society right is to set society itself right.

The awful ravages wrought by venereal diseases are but sad and practical illustrations of the Biblical axioms that "Whatsoever a man soweth, that shall he also reap," "the wages of sin is death" "the sins of the fathers are visited upon the children even unto the third and fourth generation," "no man liveth unto himself," and we might also add, so far as gonorrhea and syphilis are concerned, "no man sinneth unto himself." The man who in sowing his wild oats has contracted syphilis, later may reap its ill effects. The father who in his youthful days sowed his wild oats and later comes to realize that abortions and miscarriages or syphilitic children are the direct result of youthful excesses, believes intently that "the sins of the fathers are visited upon the children." We must set before the public both the moral and physical aspects of the problem.

It is also important that we present the medical aspect fairly and with force. When we remember that 30 per cent of all blindness is caused by gonorrhea, that 50 per cent of all sterile marriages are sterile because of gonorrhea, that 70 per cent of all abdominal operations on women are made necessary by infection due to gonorrhea, that from 70 to 80 per cent of the male population of the Republic has gonorrhea at some time, that many of these cases continue in chronic form ,that many of these men in turn infect their innocent wives, that gonorrhea inforces its primary effects with gonorrheal rheumatism, gonorrheal peritonitis, peritoneal adhesions, and general systemic infection, we come at last to a faint view of human lust being transformed into a lust that gonorrhea seems to have for ravaging the health of the individual, harming innocent wives, blinding babies and injuring the race. The bright Frenchman spoke the gospel of venery when he said that "marriage divides venereal diseases as is does the daily bread."

When we realize that 10 to 25 per cent of the race are tainted with syphilis, we get a glimpse of the truth of Osler's statement that there are more people in the average community with a leutic taint than with a tubercular taint. When we remember that about 10 per cent of all nervous diseases, 45 per cent of all abortions are caused by syphilis, we realize that to the sower must come the reaping,—and that wild oats sowed, means wild oats reaped, not only to ourselves, but also by pure wives and innocent, helpless children. Seventy per cent of all children born of syphilitic parents die, and the thirty per cent who survive are affected in one way or another, and always stunted and dwarfed and maimed in the struggle for growth and maturity. These facts would seem more awful to us were we not so familiar with them. They bring us, perhaps slowly, but surely and sadly, to the realization that modern medicine has a duty to inform as well as a duty to cure, and that the physician must teach the people so to conduct themselves as to avoid these venereal diseases. It is his province and duty to teach these facts, and thus prevent harm, as well as cure disease after harm has been wrought. Sin is the basis of the perpetuation of these diseases, and if we stop the sowing of wild oats, no man need worry about the reaping. Preventive medicine can do its perfect work solely through the instruction of the laiety by the medical profession. Preventive medicine has before it the enormous and inspiring task of teaching men to live in such thorough accord with the laws of nature and health, as that they shall cease to die of disease and begin to die of old age.

We must labor to correct four great falsehoods which involve the perpetuation of venereal woe. These false axioms and pseudo-truths are the foundation excuses for much syphilis and gonorrhea with their attendant vile evils. It is said that there is a double standard, for society is said to permit a man and to forbid the woman the illicit gratification of the sexual instinct. We must teach that there is no double standard, and that lust and its illicit gratification do not signify a double standard. We must forever impress the great truth of the world of nature, known to plant, and beast and man alike, that nature never forgives and nature never forgets. Reaping and sowing, the struggle for existence, and the survival of the fittest are parallel laws. So far as medicine and venereal diseases go, the double standard means that the sin of the man shall become in thousands of cases, the shameful disease and suffering of his innocent wife, or the reaping of his wild oats by his helpless children. Morality is the crystalization of natural law ,the true philosophy of nature, and the essence of life. Obedience to the dictates of morality simply means obedience to natural law, and the individual who lives in accord with natural law is healthy. Venereal diseases prove that health follows morality,—and disease, immorality.

The axiom of the streets teaches that "gonorrhea is no worse than a bad cold." Medicine will win a battle and render service to men, if we can inculcate in the minds of the laity the falsity of this statement, and teach that gonorrhea is a disease with far reaching and dangerous effects. The complete cure of chronic gonorrhea is a mooted question. Even if we grant it's curability, thousands of acute cases become chronic, and these play havoc with wife and mother. Each of us is an individual, and yet all the race is one, because no man liveth or sinneth unto himself. Sterile marriages and abdominal operations and blind babies and more of suffering and of woe are black and shrouded answers to the social lie that "gonorrhea is no worse than a bad cold." Knowledge of these facts implies the obligation for their dissemination to the minds of men, for knowledge is a means of safety and a road to health and purity. The physician has within himself the power to teach the ignorant to know, to inspire the weak to be strong, and to make sick folks well. The physician, of all men, knows that gonorrhea is a great deal worse than a bad cold and knowing the dangers and injuries resulting from gonorrheal infection, he can in a kind and quiet way, impress these facts as opportunity offers. Venereal diseases have offered too much of a scoffing ground, in future they offer an informing and educational ground for the relief of human suffering.

The third great falsehood that is common property on the streets teaches that illicit sexual intercourse is necessary to health. This statement is false, and to grant its truth is to grant that it is necessary to perpetuate the evils of venereal diseases. because venereal diseases will be perpetuated as long as illicit inter course is practiced and to advise illicit intercourse is to advise their perpetuation. The same sexual law governs the sexual glands in the female as in the male, and if illicit intercourse is necessary, it is necessary for the female as well as the male. Women may live to be both unmarried and healthy, happy and single. Sexual virtue and health are not incompatible, and every physician knows they are compatible. Sexual virtue never produced a disease. Sexual virtue does mean self control, but it never caused suffering or disease. Illicit intercourse means, as a rule, sooner or later, venereal disease and more or less suffering. Because it is true, and in the name of common humanity, we must teach that illicit intercourse is not necessary to health, but that it usually brings either syphilis or gonorrhea. We do not need to have periodical fits of weeping to keep the lachrymal glands in order, neither is periodic illicit intercourse necessary for the "toning" of the testicles. Nature will care for the sexual glands. Nature is a far better physician than the wild, untamed passion of the boy, or the ignorant advice of the all too ignorant or fleshly doctor of medicine. Medicine means responsibility, and we must be careful in our advice.

The sexual instinct is the most sacred of all the natural instincts. Upon its possession depends the perpetuation of the race and upon its proper care depends the sexual health of the race. The sexual act is made sacred by the Church of God and legalized by all civilized governments, in the ceremony and state of matrimony. Physiology, medicine, health, and matrimony demand the conservatism of the sexual powers in the name of the married state and the legal perpetuation of the sexual act, and the race between man and wife.

Lastly, we must teach that illicit intercourse is wrong. Every illicit act is a hidden blow at the married state and lessens the sanctity of every marriage and the fairness of every good home. If illicit intercourse is not wrong, then we can do away with marriage and home will degenerate into a by-word. Sexual chaos will supplant marriage. If illicit intercourse is not wrong for the man, it is not wrong for the woman who is the partner in the act, and if it is not wrong for the woman, then the inscrutable logic must be followed, and we must admit that it is not wrong for *any* woman. To admit this is to indict the purity of every woman, and the fairness of every home. We cannot admit the last proposition, and no man will admit the first in view of the last. Every reasonable man knows that the illicit act is contrary to the laws of God and of his better self. If we can grind these truths into the minds and hearts of the boys of this generation and the generation to come, we shall live in a purer, healthier and happier world.

The presence of the negro in the Southern States increases the importance of purity in sexual matters. The balance between two separate races living in the same country is delicate at best, and lust and licentiousness may unsettle the balance. The mulatto is not the offspring of Southern chivalry, but of a passion that has forgotten its chivalry. It is conservatively estimated that 75 per cent of negroes in the cities and towns of the Southern States are tainted with syphilis, and about 95 per cent or more have gonorrhea at some time. There must be created a sentiment that shall be not merely a sentiment, but a working principle, against the cohabiting of the races. We must develop, somehow, a firm molding of the wildest and most passionate boy into a perfect obedience to a new, unwritten, and hitherto unmentioned law,—that no Southern boy shall cohabit with any black woman, and that Caucasian whiteness shall not be stained, no matter how black the night, or how lonely the place, with the shame and stench of an Ethiopian partner. And finally, all Caucasian blood is blue blood, and should be pure blood, because to it has come the heritage of all ages, and the ages back of it and the God above it, demanded that Anglo-Saxon blood be the perfect fruit of a fruitful time, and pure.

834 Candler Bldg.

